# ALTERED GENE EXPRESSION PROFILE OF AGING BONE MARROW MESENCHYMAL STEM CELLS

**DOI:** 10.1093/geroni/igad104.2479

**Published:** 2023-12-21

**Authors:** Yun Su, Jie Chen, Carlos Isales, Xingming Shi

**Affiliations:** Augusta University, Augusta, Georgia, United States; Augusta University, Augusta, Georgia, United States; Augusta University, Augusta, Georgia, United States; Augusta University, Augusta, Georgia, United States

## Abstract

Aging is a complex process that affects many physiological systems, including bone metabolism. To gain insight into the molecular mechanisms underlying age-related bone loss, we conducted RNA-seq analysis of mesenchymal stem cells (MSCs) isolated from the bone marrow of 6-month and 24-month-old C57Bl/6 mice. We identified 5857 differentially expressed genes, out of 19,324 genes, with an absolute fold change greater than 1.4 and FDR q-value less than 0.05. These genes are involved in a variety of biological processes, including metabolism, immune response, and signal transduction. KEGG pathway enrichment analysis revealed 28 significantly enriched pathways, suggesting that multiple pathways contribute to age-related bone loss. Our findings provide a foundation for future studies aimed at elucidating the molecular mechanisms underlying age-related bone loss and identifying potential therapeutic targets for the prevention and treatment of osteoporosis.

